# Origin of malaria cases: a 7-year audit of global trends in indigenous and imported cases in relation to malaria elimination

**DOI:** 10.3402/gha.v8.29133

**Published:** 2015-10-07

**Authors:** Mar Velarde-Rodríguez, Rafael Van den Bergh, Cristin Fergus, Aina Casellas, Sergi Sanz, Richard Cibulskis, Andrew R. Ramsay, Karen Bissell, Rony Zachariah

**Affiliations:** 1 Barcelona Institute for Global Health, Barcelona, Spain; 2 Brussels Operational Centre, Médecins sans Frontières, Luxembourg, Luxembourg; 3 Global Malaria Programme, World Health Organization, Geneva, Switzerland; 4 Special Programme for Research and Training in Tropical Diseases, World Health Organization, Geneva, Switzerland; 5 International Union against Tuberculosis and Lung Disease, Paris, France

**Keywords:** malaria, infection origin, imported case, indigenous case, elimination, prevention of reintroduction, eradication, operational research

## Abstract

**Background:**

Countries in the different stages of pre-elimination, elimination, and prevention of reintroduction are required to report the number of indigenous and imported malaria cases to the World Health Organization (WHO). However, these data have not been systematically analysed at the global level.

**Objective:**

For the period 2007 to 2013, we aimed to report on 1) the proportion of countries providing data on the origin of malaria cases and 2) the origin of malaria cases in countries classified as being in the stages of pre-elimination, elimination and prevention of reintroduction.

**Design:**

An observational study using annual data reported through routine health information systems to the WHO Global Malaria Programme between 2007 and 2013.

**Results:**

For all countries classified as being in pre-elimination, elimination, and prevention of reintroduction in the year 2013, there has been a substantial decrease in the total number of indigenous malaria cases, from more than 15,000 cases reported in 2007 to less than 4,000 cases reported in 2013. However, the total number of imported malaria cases has increased over that time period, from 5,600 imported cases in 2007 to approximately 6,800 in 2013.

**Conclusions:**

Vigilant monitoring of the numbers of imported and indigenous malaria cases at national and global levels as well as appropriate strategies to target these cases will be critical to achieve malaria eradication.

Malaria elimination is defined as the interruption of local transmission of a specified malaria parasite species in a defined geographical area as a result of deliberate efforts (1). According to the World Health Organization (WHO), the path to malaria-free status is characterised by four progressive programmatic stages: control, pre-elimination, elimination, and prevention of reintroduction (Box 1) (1, 2).

**Box 1:** Definitions of malaria control, pre-elimination, elimination, and prevention of reintroduction, and malaria-free status (1, 2)

**Control:** The reduction of the malaria disease burden to a level at which it is no longer a public health problem. **Pre-elimination:** The annual incidence of malaria in the district with the highest number of cases being less than five cases per 1,000 population. **Elimination:** Interruption of local transmission (reduction to zero incidence) of a specified malaria parasite in a defined geographic area as a result of deliberate efforts. Continued measures to prevent reestablishment of transmission are required. **Prevention of reintroduction**: 1) Recently, endemic country with zero local transmission for at least 3 years or 2) country on the WHO Register or Supplementary List that has ongoing local transmission.^a^ **Malaria-free status:** An area in which there is no continuing local mosquito-borne malaria transmission and the risk for acquiring malaria is only limited to introduced cases.^b^

a Ongoing local transmission=2 consecutive years of local *P. falciparum* malaria transmission or 3 consecutive years of local *P. vivax* malaria transmission in the same locality or otherwise epidemiologically linked.

b Introduced: A case contracted locally, with strong epidemiological evidence linking it directly to a known imported case.

Health professionals in countries that are in the elimination and prevention of reintroduction stages are required to notify each confirmed case to the National Malaria Control Programme, institute treatment, and conduct an investigation to determine the origin of infection. This epidemiological investigation determines whether a case is indigenous or imported.

An indigenous case is any case contracted locally, without strong evidence of a direct link to an imported case (3). An imported case is a case due to mosquito-borne transmission and acquired outside the country (3).

An investigation form or questionnaire is generally used to carry out the epidemiological investigation. This form includes a few questions about recent travel history to a foreign endemic country. To assess whether the case is indigenous or imported, there are a number of factors to be considered such as the time between returning from an endemic country and the detection of malaria infection (3). In addition, the malaria programmes need to consider which species is causing the infection because *Plasmodium vivax* relapses can occur within 3 years of the primary infection (3).

Evidence from a number of countries moving to elimination has shown that despite a decrease in the number of indigenous cases, the number of imported cases has risen due to an increasing movement of people between countries (4–7). Imported cases may be less amenable to malaria interventions than indigenous cases because they may include a relatively mobile population. It is important to accurately monitor the number of imported cases because they play a key role in re-establishing and sustaining malaria transmission (6, 8–11).

The WHO requests countries in stages of malaria pre-elimination, elimination, and prevention of reintroduction phases to systematically report on the origin (indigenous or imported) of cases. This paper analyses 1) the proportion of countries providing data on the origin of malaria cases and 2) the origin of malaria cases in countries classified as being in the stages of pre-elimination, elimination and prevention of reintroduction.

## Methods

### Study design

An observational study using annual data reported through routine health information systems to the WHO Global Malaria Programme (GMP) between 2007 and 2013.

### Setting and study population

Country-level information on malaria is submitted annually to WHO regional offices and then to GMP in Geneva, Switzerland. Validation is performed at country level as well as by the WHO regional and global levels. The results are published annually in the *World Malaria Report*.

Aggregate data on confirmed malaria cases in countries in pre-elimination, elimination, and prevention of reintroduction were included in this study.

Countries in pre-elimination, elimination, and prevention of reintroduction can transition between these categories within a few years. For example, a country that was classified as being in elimination in the year 2007 could be classified as being in prevention of reintroduction in the year 2013.

### Data collection and analysis

Data were extracted from the malaria database available at the GMP and analysed using Microsoft Excel. Proportions were used to express the trends of malaria cases by origin.

### Ethics

The study was approved by the Ethics Advisory Group of the International Union Against Tuberculosis and Lung Disease, Paris, France. As this was a study of routinely collected aggregate data, informed consent did not apply.

## Results

At present, there are 97 countries with ongoing malaria transmission; this includes 78 countries in the control phase, 10 countries in the pre-elimination stage, and 9 countries in the elimination phase. An additional seven countries are classified as being in the stage of prevention of reintroduction (2). Country classification by stage of malaria elimination in 2013 is shown in Table 1.

**Table 1:** Classification of countries by stage of elimination, December 2014

Region	Pre-elimination	Elimination	Prevention of reintroduction	Certified as malaria-free since 2000
AFR	Cape Verde	Algeria		
AMR	Belize Costa Rica Ecuador El Salvador Mexico Paraguay	Argentina		
EMR		Iran (Islamic Republic of) Saudi Arabia	Egypt Iraq Oman Syrian Arab Republic^a^	Morocco – 2010 United Arab Emirates – 2007
EUR		Azerbaijan Tajikistan Turkey	Georgia Kyrgyzstan Uzbekistan	Turkmenistan – 2010 Armenia – 2011
SEAR	Bhutan Democratic People's Republic of Korea	Sri Lanka		
WPR	Malaysia	Republic of Korea		

a Limited information is available from the Syrian Arab Republic. *Source:* World Malaria Report 2014.

**Fig. 1:**
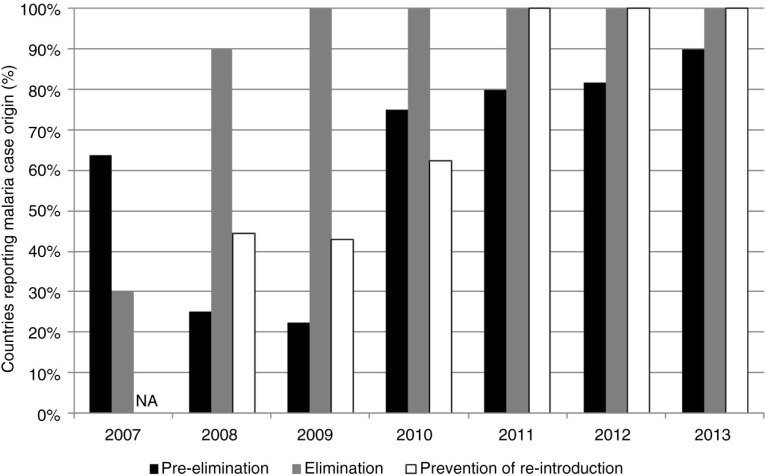
Proportion of countries reporting the origin of malaria cases between 2007 and 2013, by stage of malaria elimination. NA=Not applicable

A steadily increasing proportion of countries that were classified in stages of malaria pre-elimination, elimination, and prevention of reintroduction between 2007 and 2013 has been reporting indigenous and imported cases to WHO (Fig. 1). Over the past 3 years, all countries in elimination and prevention of reintroduction have reported the origin of malaria cases to WHO.

**Fig. 2:**
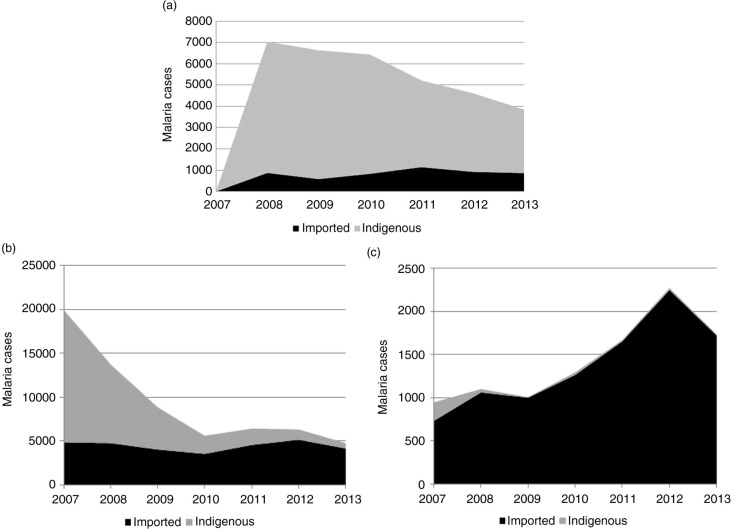
Total number of imported and indigenous malaria cases reported by countries in pre-elimination, elimination and prevention of reintroduction, 2007–2013. (a) Pre-elimination.^a^ (b) Elimination. (c) Prevention of reintroduction. ^a^For countries in pre-elimination, data on indigenous and imported cases was not available for year 2007.

For all countries classified as being in pre-elimination in 2013 (Fig. 2a), there were no data available on indigenous and imported cases in the year 2007. The total number of indigenous malaria cases has decreased from more than 6,000 in 2008 to less than 3,000 in 2013. The number of imported malaria cases has remained fairly stable over the same period; in 2013, there were 865 imported cases in countries classified in pre-elimination.

For all countries classified as being in elimination in 2013 (Fig. 2b), the total number of indigenous malaria cases decreased from approximately 15,000 in 2007 to less than 700 in 2013. For these countries, however, the total number of imported cases did not vary much over that period. In total, 4,182 imported malaria cases were reported in elimination countries in 2013.

For all countries classified as being in prevention of reintroduction in 2013 (Fig. 2c), there have been no indigenous cases reported since 2009. However, the number of imported cases doubled in 6 years, from 700 in 2007 to more than 1,500 in 2013. It is important to note that the peak of imported cases in 2012 was largely due to Oman, which has been battling small malaria outbreaks since 2007 related to importation of parasites.

**Fig. 3:**
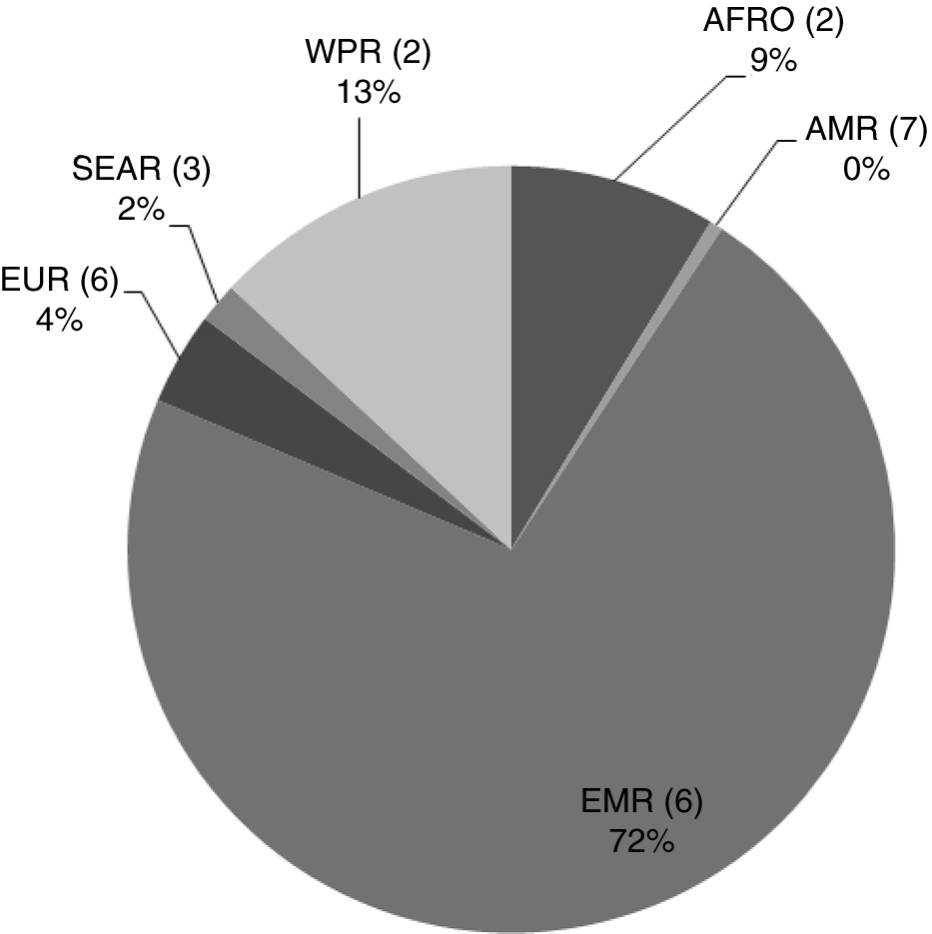
Proportion of imported malaria cases by WHO region, 2013. AFR, African Region; AMR, Region of the Americas; EMR, Eastern Mediterranean Region; EUR, European Region; SEAR, South-East Asia Region; WPR, Western Pacific Region. The numbers in brackets indicate the total number of countries in pre-elimination, elimination, and prevention of reintroduction in each of the regions.

Globally, 6,771 imported malaria cases were reported to WHO in 2013 (Fig. 3). In the Eastern Mediterranean Region, there were six countries in elimination and prevention of reintroduction; they reported 72% of these imported cases. The European Region also had six countries in elimination and prevention of reintroduction, and in the Americas Region, there were seven countries in pre-elimination and elimination. However, these regions together accounted for less than 5% of imported cases in the year 2013.

In both the Western Pacific Region and the African Region, there were two countries in pre-elimination and elimination stages; they reported 13% and 9% of the imported cases, respectively. The South-East Asia Region had three countries in pre-elimination and elimination stages, however, only two of these countries reported data on malaria origin to WHO, and these countries accounted for 2% of the total imported cases in the year 2013.

## Discussion

In the context of the Global Technical Strategy for Malaria 2016–2030, which was adopted by the World Health Assembly in May 2015 and aims to accelerate progress towards malaria elimination, monitoring the numbers of imported and indigenous malaria cases is important. This study shows that the proportion of countries reporting data on geographic origin of malaria cases has progressively improved, which is encouraging. In terms of reporting, a number of issues merit discussion.

First, should only countries in pre-elimination, elimination, and prevention of reintroduction report the origin of malaria cases, or should this be extended to all countries in the control phase? Of note, there are currently 78 countries classified as being in the control phase that are currently not required to report data on indigenous and imported malaria cases to WHO. In these countries, one important factor to consider would be what proportion of malaria cases is likely to be imported. Should WHO also keep records of imported malaria cases in countries that have recently been certified as malaria-free? It would seem logical if we want to keep updating the global map of malaria.

Second, considering the burden in countries in the elimination and prevention of reintroduction stages, imported cases of malaria need particular attention. As imported malaria cases are one of the main threats to malaria elimination, better indicators for imported cases are clearly needed. Currently, the imported cases are counted in the countries where they are detected, and they are not counted as local transmission in the countries they come from. This represents a challenge in the epidemiological monitoring of malaria transmission worldwide.

Third, national surveillance systems should always collect epidemiological information for imported cases, including the country of origin as well as the *Plasmodium* species causing the infection. Currently, national malaria programmes rely on a patient's travel history to ascertain where the infection is acquired (1). If the infected individual lives in an area where there is local transmission, then the case will be classified as indigenous unless it can be shown that he/she could not have acquired the infection locally. At the moment, more accurate tools for case investigation are not used in the field. Molecular methods to distinguish the origin of infection are currently being assessed (12), and if they can be provided at affordable costs, they may be a game changer in identifying the geographic origin of infection.

Fourth, remarkable progress has been achieved towards malaria elimination in a number of countries over the past years. Various regional initiatives have been introduced to support efforts to eliminate malaria such as Asia Pacific Malaria Elimination Network, the Elimination Eight Regional Initiative, the Greater Mekong Subregion, the Mesoamerica and Hispaniola Regional Initiative, and the Tashkent Declaration (13). These regional initiatives should continue supporting collaborative efforts in border areas and regions of high population mobility, where imported malaria is of concern in order to reduce the number of both imported and indigenous malaria cases (12–15).

In conclusion, despite an encouraging decrease in the number of indigenous malaria cases over the past 7 years, the number of imported malaria cases has not seen an equivalent reduction in countries moving towards malaria elimination. This issue merits focused attention if we are to continue to shrink the malaria map and hold on to what has already been achieved.

Monitoring the numbers of imported and indigenous malaria cases is going to be critical as more countries move to malaria elimination over the next years. Robust epidemiological data and better indicators for imported cases will be needed to evaluate the progress made at country, regional, and global levels. Regional initiatives have a key role and should continue their support to malaria elimination.
